# Anterior Displacement of Lamina Cribrosa during Valsalva Maneuver in Young Healthy Eyes

**DOI:** 10.1371/journal.pone.0159663

**Published:** 2016-07-21

**Authors:** Yong Woo Kim, Michael J. A. Girard, Jean Martial Mari, Jin Wook Jeoung

**Affiliations:** 1 Department of Ophthalmology, Armed Forces Busan Hospital, Busan, Korea; 2 Department of Biomedical Engineering, National University of Singapore, Singapore, Singapore; 3 Singapore Eye Research Institute, Singapore, Singapore; 4 University of French Polynesia, Tahiti, French Polynesia; 5 Department of Ophthalmology, Seoul National University Hospital, Seoul National University College of Medicine, Seoul, Korea; University of Florida, UNITED STATES

## Abstract

**Purpose:**

To investigate lamina cribrosa (LC) displacement during the Valsalva maneuver in young healthy eyes using enhanced depth imaging (EDI) spectral-domain optical coherence tomography (SD-OCT).

**Methods:**

Forty-eight eyes of 48 young healthy volunteers (age range: 20–34 years) underwent intraocular pressure (IOP) measurement by Goldmann applanation tonometry as well as Cirrus HD-OCT scans before and during the Valsalva maneuver. The optic nerve head (ONH) parameters (average retinal nerve fiber layer thickness, rim area, disc area, average C/D ratio, vertical C/D ratio, cup volume), anterior LC depth (LCD), subfoveal and peripapillary choroidal thickness, and neural canal opening diameter were measured on compensated OCT and compared during Valsalva challenge. The subjects were asked to take a five-minute break after each Valsalva maneuver.

**Results:**

During the Valsalva maneuver, the IOP significantly increased, from 12.7 ± 3.0 mmHg to 16.0 ± 3.2 mmHg (*P* < 0.001), while the LCD sharply decreased, from 463.4 ± 118.8 μm to 427.3 ± 106.4 μm (*P* < 0.001). The subfoveal choroidal thickness (300.7 ± 90.6 vs. 309.6 ± 93.5 μm), peripapilllary choroidal thickness (152.2 ± 55.4 vs. 150.8 ± 49.3 μm), neural canal opening diameter (1651.8 ± 204.2 vs. 1651.0 ± 217.6 μm), and all of the ONH parameters did not change significantly (all *P* > 0.05).

**Conclusions:**

The Valsalva maneuver induced anterior displacement of the LC, but did not alter the choroidal thickness or ONH morphology. The data describe the positional characteristics of the LC in response to the Valsalva maneuver in young healthy eyes.

## Introduction

Posterior displacement of the lamina cribrosa (LC) caused by increased intraocular pressure (IOP) plays a prominent role in the pathogenesis of glaucoma.[1–3] A posteriorly displaced LC can lead to mechanical and vascular damage to the optic nerve head (ONH), including the ganglion cell axons.[1] However, glaucomatous eyes even within the normal range of IOP, a condition known as normal-tension glaucoma (NTG), remain at issue in the ongoing quest to discover additional causal factors of glaucoma development.

Recently, IOP and cerebrospinal fluid (CSF) pressure dynamics as they impact on the LC have been considered to be a key factor in glaucoma development.[4–7] Trans-laminar pressure difference (TLPD; the difference between IOP and CSF pressure) and trans-laminar pressure gradient (TLPG; TLPD divided by LC thickness) recently have been suggested as possible factors in the pathogenesis of NTG.[5] CSF pressure in NTG eyes is known to be lower than in high-tension glaucoma eyes or healthy controls.[8, 9] In addition, axonal damage and optic neuropathy can be induced by experimental reduction of CSF pressure in monkey eyes.[10]

In this light, Zhang et al.[11] investigated in-vivo structural ONH change under the opposite circumstances: increased CSF pressure resulting from the sustained Valsalva maneuver in non-glaucomatous eyes. The subjects with lumbar puncture demonstrated that the Valsalva maneuver induced a decrease or reversal of TLPD. Another group of subjects revealed an inward movement of the ONH, a decrease of cup volume and cup-to-disc (C/D) ratio and an increase of neuroretinal rim volume during the Valsalva maneuver. The position of the LC in relation to TLPD dynamics, however, has yet to be investigated.

We hypothesized that the Valsalva maneuver reverses TLPD by increasing CSF pressure, which might lead to anterior displacement of the LC. The purpose of the present study was to investigate LC positional change during the Valsalva maneuver.

## Materials and Methods

### Study Subjects

The participants in this study comprised 48 young healthy volunteers who visited the Armed Forces Busan Hospital for health screening checkup. They met the eligibility criteria and provided written informed consent to participate. This study was approved by the Seoul National University Hospital and Armed Forces Medical Command Institutional Review Board and followed the tenets of the Declaration of Helsinki.

Subjects’ detailed medical histories were taken, after which they underwent comprehensive ophthalmic examinations including best-corrected visual acuity assessment, slit-lamp biomicroscopy, refraction, gonioscopy, Goldmann applanation tonometry, and dilated fundus examination. Central corneal thickness (CCT) was measured by SD-OCT (anterior segment scan mode, Cirrus HD-OCT 5000, Carl Zeiss Meditec, Dublin, CA) via built-in caliper system (software version 6.0; Carl Zeiss Meditec).

The IOP was measured twice before the standardized Valsalva maneuver and then once during it. The baseline IOP value was defined as the mean of the two measurements before the standardized Valsalva maneuver.

None of the eyes had glaucomatous optic disc changes (e.g., notching, rim thinning, RNFL defect) on fundus examination. SD-OCT scans showed peripapillary RNFL and macular ganglion cell-inner plexiform layer (GCIPL) thicknesses within the normal ranges.

The present study excluded subjects with (1) a history of refractive surgery, (2) a history of ocular trauma, (3) a history of systemic or ocular infection, (4) a history of systemic diseases including hypertension and diabetes, (5) intraocular pressure (IOP) > 21 mmHg, (6) SD-OCT scan signal strength < 7, or (7) unclear visibility of more than one-quarter of the anterior LC surface of the neural canal opening diameter. Only one eye was randomly selected for the analysis.

### Measurement protocol with Standardized Valsalva maneuver

The subjects were asked to exhale into a mouthpiece connected to a handheld differential pressure meter (OMEGA HHP-801TM; OMEGA Engineering Inc., Connecticut, USA). They were instructed to maintain the expiratory pressure at a minimal level of 30 mmHg for over 15 seconds. After each Valsalva maneuver, the subjects were directed to take a five-minute break.

### Spectral-Domain Optical Coherence Tomography Imaging

All of the subjects were scanned with the Cirrus HD-OCT 5000. The optic disc scan was performed as follows: 200 × 200 optic disc cube scan, 5-HD line scans (6 mm length) centered to optic disc, and 1-HD line scan (9 mm length) aligned to axis connecting fovea and center of optic disc. The HD-line scans were performed in the enhanced depth imaging (EDI) mode. All of the optic disc scans were performed in a sitting position, first as a baseline and then with the subject sustaining the standardized Valsalva maneuver. The subjects rested for five minutes after each Valsalva maneuver prior to initiation of the next scan. A 200 × 200 macular cube scan was performed as a baseline measurement to obtain the macular ganglion cell-inner plexiform layer (GCIPL) thickness. The average peripapillary RNFL thickness and the following ONH parameters were automatically measured with the built-in analysis algorithm (software version 6.0; Carl Zeiss Meditec): rim area, disc area, average C/D ratio, vertical C/D ratio, and cup volume.

### Measurement of Anterior Lamina Cribrosa Depth, Neural Canal Opening Diameter, and Subfoveal and Peripapillary Choroidal Thicknesses

To enhance the LC and choroid visibility, adaptive compensation was performed on all of the optic disc scan images according to the relevant protocols (contrast exponent = 2, threshold exponent = 6).[12–14] All of the measurements were performed using ImageJ software (developed by Wayne Rasband, National Institutes of Health, Bethesda, MD; available at http://imagej.nih.gov/ij/). Anterior LC depth (LCD) was defined as the maximal vertical distance between the reference plane connecting Bruch’s membrane openings (BMO) and the anterior LC surface. Neural canal opening diameter was defined as the distance between the two terminations of BMO. Choroidal thickness was defined as the vertical distance between the outer border of the retinal pigment epithelium and the inner surface of the sclera. The LCD and neural canal opening diameter were measured from five HD-line scans, and only the central three of them were used for the analysis. The subfoveal choroidal thickness and peripapillary choroidal thickness (the point 250 μm temporally from the termination of the BMO) were measured simultaneously from a 1-HD line scan. The measurements were performed by an experienced ophthalmologist (Y.W.K.) who was masked to each subjects’ clinical information.

### Data Analysis

To determine the interobserver reproducibility of the LCD and choroidal thickness measurements, 10 randomly selected SD-OCT scans were evaluated by two independent examiners (Y.W.K, J.W.J.), and the intraclass correlation coefficient (ICC) was calculated; additionally, the intersession variability of the LCD was obtained from 15 healthy volunteers. The ICC and intersession standard deviation (SD) of the scans, which had been repeated at 30 minute intervals, were calculated. The LCD change was deemed statistically significant when it exceeded 1.96-times the intersession SD, since this corresponds to the 95% confidence interval for the true value of the measurement.[15–17]

The continuous variables were compared using a paired t-test. Statistical analyses were performed with the Statistical Package for Social Sciences version 21.0 for Windows (SPSS, Inc., Chicago, IL). The data were obtained, and are presented in this paper, as mean ± standard deviations, the level of statistical significance having been set at *P* < 0.05.

## Results

### Baseline Characteristics

A total of 50 young healthy volunteers were initially recruited. Two subjects were excluded because of unclear visibility of anterior LC surface. Finally, 48 young healthy volunteers, eight of whom were female, were included in the study. The mean age was 25.3 ± 5.1 years (range: 20–34). Table 1 provides summarized demographics on the subjects.

**Table 1:** Demographics of the subjects.

Variables	Subjects (*n* = 48)
Age, yr	25.6 ± 5.4 (20–34)
Male / Female	40 / 8
Baseline IOP, mmHg	12.7 ± 3.0
Average RNFL thickness, μm	98.4 ± 10.3
Average GCIPL thickness, μm	82.7 ± 4.6
CCT, μm	529.1 ± 39.4

Mean ± standard deviation, only right eye of the subjects were included.

RNFL = retinal nerve fiber layer, GCIPL = ganglion cell-inner plexiform layer, CCT = central corneal thickness.

### Optic Nerve Head Parameter Change during Standardized Valsalva Maneuver

The IOP at baseline (12.7 ± 3.0 mmHg) significantly increased during the standardized Valsalva maneuver (16.0 ± 3.2 mmHg, *P* < 0.001). This increase was evident in 47 of the 48 eyes (97.9%). The IOP of only one eye (2.1%) decreased (2.0 mmHg). There was no significant change of average RNFL thickness (98.4 ± 10.3 vs. 97.0 ± 10.1 μm, *P* = 0.11) and optic nerve head parameters during the standardized Valsalva maneuver (Table 2).

**Table 2:** Optic nerve head parameters change during standardized Valsalva maneuver.

Variables	Baseline (*n* = 48)	During Valsalva (*n* = 48)	*P*-value (*)
Average RNFL thickness, μm	98.4 ± 10.3	97.0 ± 10.1	0.11
Rim area, mm^2^	1.28 ± 0.16	1.26 ± 0.16	0.10
Disc area, mm^2^	1.87 ± 0.38	1.85 ± 0.38	0.09
Average cup-to-disc ratio	0.52 ± 0.13	0.52 ± 0.14	0.63
Vertical cup-to-disc ratio	0.49 ± 0.13	0.49 ± 0.13	0.93
Cup volume, mm^3^	0.196 ± 0.151	0.193 ± 0.146	0.28

Mean ± standard deviation,

* Comparison performed using paired t-test.

### Anterior LC Depth Change during Standardized Valsalva Maneuver

**Fig 1:**
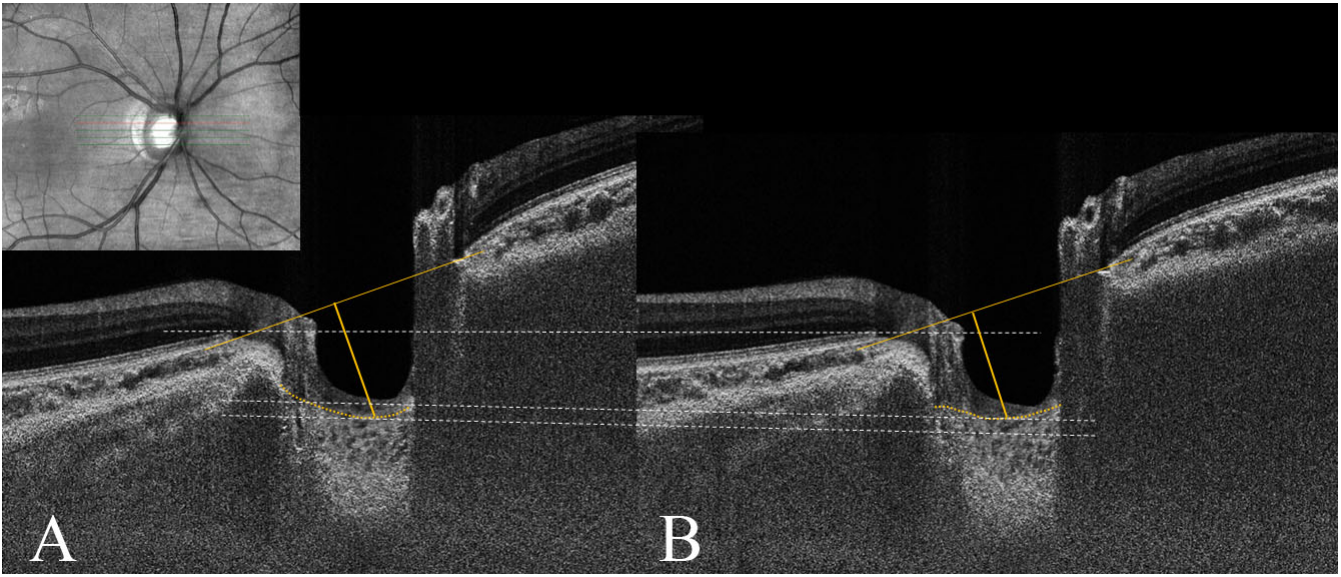
Anterior displacement of lamina cribrosa (LC) during standardized Valsalva maneuver. Enhanced depth imaging (EDI) optic disc scan of 26-year-old healthy male (A) at baseline and (B) during standardized Valsalva maneuver. Note that adaptive compensation was performed to enhance lamina cribrosa (LC) visibility. The anterior LC depth (LCD) was defined as the maximal distance between the reference plane connecting the Bruch’s membrane openings (BMO) and the anterior LC surface. The LCD decreased from 607 μm at baseline to 563 μm during the Valsalva maneuver, while the intraocular pressure (IOP) increased from 14 mmHg at baseline to 17 mmHg.

**Fig 2:**
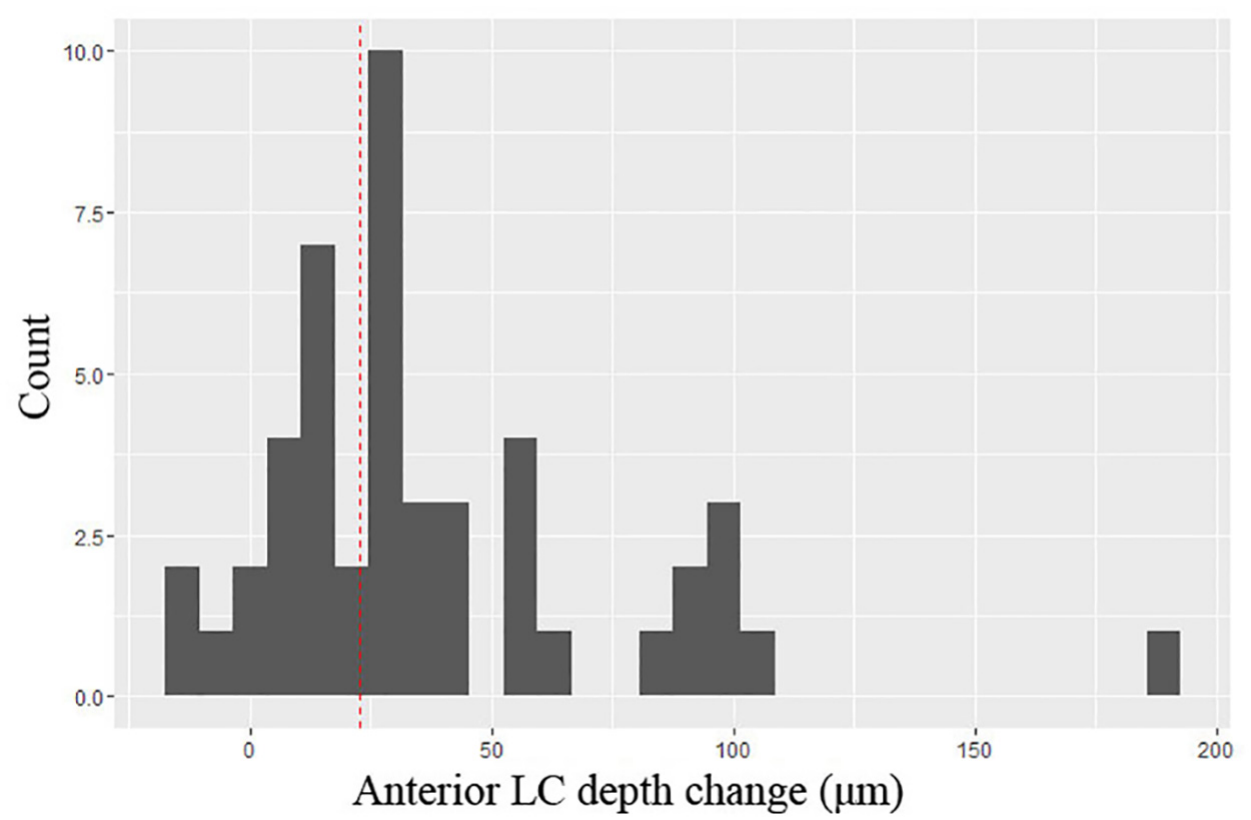
Distribution of anterior lamina cribrosa depth change during standardized Valsalva maneuver. The histogram presents the distribution of the anterior LC depth change during the standardized Valsalva maneuver. Twenty-nine eyes (60.4%) showed significant anterior displacement of the LC during the standardized Valsalva maneuver. Only three eyes (6.3%) showed posterior displacement (10.0–17.0 μm). The vertical red dotted line depicts the 1.96-times intersession SD of the OCT measurements (22.5 μm).

The interobserver reproducibility of the LCD measurements was excellent (ICC = 0.993 and 95% CI = 0.972–0.998, *P* < 0.001). The intersession reproducibility of the LCD also was excellent (ICC = 0.997, 95% CI = 0.990–0.999). The 1.96-times intersession SD was 22.5 μm. Twenty-nine eyes (60.4%) showed significant anterior displacement of the LC during the standardized Valsalva maneuver. Only three eyes (6.3%) showed posterior displacement (10.0–17.0 μm) (Figs 1 and 2). The mean LCD at baseline (463.4 ± 118.8 μm) significantly decreased during the standardized Valsalva maneuver (427.3 ± 106.4 μm, *P* < 0.001). However, the neural canal opening diameter did not change significantly (1651.8 ± 204.2 vs. 1651.0 ± 217.6 μm, *P* = 0.94) (Table 3).

**Table 3:** Anterior lamina cribrosa depth, neural canal opening diameter, and choroidal thickness change during standardized Valsalva maneuver.

Variable (μm)	Baseline (*n* = 48)	During Valsalva (*n* = 48)	*P*-value (*)
Anterior LC depth	**463.4 ± 118.8**	**427.3 ± 106.4**	**< 0.001**
NCO diameter	1651.8 ± 204.2	1651.0 ± 217.6	0.94
Subfoveal choroidal thickness	300.7 ± 90.6	309.6 ± 93.5	0.18
Peripapillary choroidal thickness	152.2 ± 55.4	150.8 ± 49.3	0.68

Mean ± standard deviation, statistically significant values are shown in bold,

*Comparison performed using paired t-test.

NCO = neural canal opening.

### Choroidal Thickness Change during Standardized Valsalva Maneuver

**Fig 3:**
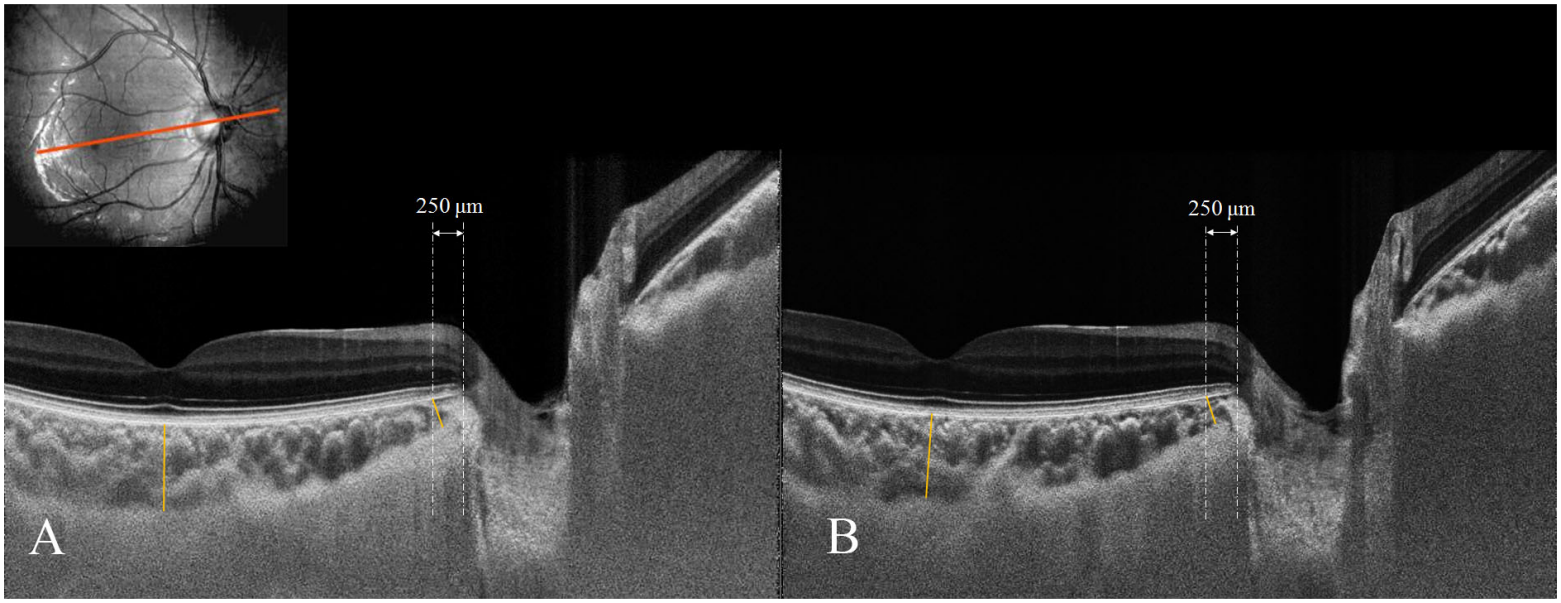
Choroidal thickness change during standardized Valsalva maneuver. Enhanced depth imaging (EDI) scan aligned to axis connecting fovea and center of optic disc in 22-year-old male. Note that adaptive compensation was performed to enhance choroid visibility. Choroidal thickness was defined as the vertical distance between the outer border of the retinal pigment epithelium and the inner surface of the sclera. The peripapillary chorodidal thickness was measured at the point 250 μm temporally from the termination of the Bruch’s membrane openings (BMO). The subfoveal choroidal thickness (A) was 336 μm at baseline and 324 μm during the standardized Valsalva maneuver. The peripapillary choroidal thickness (B) was 154 μm at baseline and 146 μm during the standardized Valsalva maneuver.

The interobserver reproducibility of the choroidal thickness measurement was excellent (ICC = 0.993, 95% CI = 0.967–0.998, *P* < 0.001). The baseline subfoveal choroidal thickness did not change significantly during the Valsalva maneuver (300.7 ± 90.6 vs. 309.6 ± 93.5 μm, *P* = 0.18). The baseline peripapillary choroidal thickness (152.2 ± 55.4 μm) also showed no significant change (150.8 ± 49.3 μm, *P* = 0.68) (Fig 3, Table 3).

## Discussion

The present study investigated LC positional change during the standardized Valsalva maneuver. Over 60% of the young healthy eyes showed significant anterior displacement of the LC, though there were no significant changes in the ONH parameters or choroidal thickness.

The anterior displacement of the LC might have resulted from TLPD reversal during the Valsalva maneuver. IOP variance during the Valsalva maneuver has been investigated in the literature, though its precise mechanism remains unclear.[18–21] Raised intrathoracic pressure during the Valsalva maneuver reduces venous return via the superior and inferior vena cava, thus inducing engorgement of the jugular, orbital, and vortex veins.[22] IOP rise is hypothesized to be due to choroidal engorgement or increased episcleral venous pressure.[18, 23] However, this cannot explain documented cases of eyes showing IOP reduction during the Valsalva maneuver.[11, 18, 21, 24] The present study demonstrated an IOP increase of 3.3 ± 1.7 mmHg (range: –2–9 mmHg) during the Valsalva challenge, including only one case with IOP decrease, which results are comparable to the previous reports.[11, 18–21, 23]

Meanwhile, Zhang et al.[11] recently demonstrated that during the Valsalva maneuver, increased lumbar CSF pressure was significantly higher than increased IOP. They also, though indirectly, established that TLPD can be reversed during the Valsalva maneuver. Although the current study did not measure the CSF pressure (due to the ethical issue raised in performing invasive procedures on healthy volunteers), its findings can be considered to be analogous and complementary to Zhang et al.’s report. It may be argued that the LC can be anteriorly displaced due to scleral stretch caused by increased IOP during the Valsalva maneuver.[25] If this occurred, a considerable accompanying increase of neural canal opening diameter and alteration of ONH structures would be expected. However, we did not detect any such changes.

In this study, the subfoveal and peripapillary choroidal thicknesses did not change significantly during the Valsalva maneuver. Falcao et al.[26] similarly found no significant changes of subfoveal choroidal thickness during the Valsalva maneuver in nine young healthy volunteers. These findings though are inconsistent with Schuman et al.’s report, which presented evidence of significant ciliary-body thickness increase (up to 70 μm) during wind instrument playing.[24] The discrepancy can be explained by the different vascular responses to the Valsalva maneuver in different parts of the uvea, which effect asymmetric (anterior and posterior) choroidal change.[26] In any case, differences in expiratory effort (Valsalva maneuver vs. high-resistance wind instrument playing) might have resulted in inconsistent findings, which possibility should be carefully considered.

Schuman et al.[24] investigated the hazard of playing high-resistance wind instruments with respect to glaucoma development, reporting that such musicians showed significantly greater visual field loss compared with other musicians. The findings of the present study are inconsistent with these. This fact however must be treated with caution, as the participants in the present study were younger healthy individuals who naturally had not performed the Valsalva maneuver frequently. The LC, furthermore, is known to remodel physiologically in response to aging.[27–29] In fact, alteration of tissue characteristics with age can change the LC response during Valsalva challenge. Certainly, the current findings should not be generalized to older or glaucomatous eyes that can have different LC responsiveness to TLPD. Further investigation with subjects representative of this population presumably will clarify the relationship between LC displacement and TLPD.

The present study found that ONH parameters did not change significantly during the Valsalva maneuver. This result is inconsistent with Zhang et al.’s report, which indicated inward movement of the ONH.[11] This disparity might have been caused by 1) differences in expiratory efforts and duration during the Valsalva maneuver, despite the use of an expiratory manometer, 2) differences in the range of IOP change during Valsalva challenge, 3) the difference in the devices (SD-OCT vs. confocal scanning laser tomography) utilized to detect ONH change, or 3) differences (e.g. age) between the study populations.

This study has some shortcomings. First, the BMO plane was used as the reference for the LCD measurement, though the location of the BMO is known to vary according to choroidal thickness.[30–32] Even though our subfoveal and peripapillary choroidal thickness measurements showed no change during the Valsalva maneuver, if choroidal engorgement during Valsalva challenge increased the choroidal thickness, the LCD might have been increased. This would mean that the LCD decrease observed during the Valsalva maneuver was smaller than the actual change, which actually would strengthen our hypothesis. Second, we did not measure the CSF pressure change during the Valsalva maneuver. Thus, the data cannot be generalized to dose-response relationship between TLPD and LC displacement but rather only to determine whether the LC is displaced or not in consequence of the Valsalva maneuver. Also, maintaining at least a certain consistency of expiratory pressure during Valsalva challenge would not guarantee precise control of orbital CSF pressure. In that precise in-vivo measurement of orbital CSF pressure is practically impossible, well controlled quantitative analysis is very difficult to achieve clinically. Nevertheless, as pilot study data, the present results can serve as a reference for future prospective studies investigating the relationship between the Valsalva maneuver and LC displacement.

In conclusion, the Valsalva maneuver commonly induces anterior displacement of the LC in young healthy eyes, as confirmed by SD-OCT imaging. The data describe the positional characteristics of the LC in response to IOP and CSF pressure dynamics in young healthy eyes.
